# Coming of Age

**Published:** 1887-10-22

**Authors:** 


### COMING OF AGE.

He came of age! How proud we were to greet
Our boy, who touched his manhood on that day;
But ere the day reached evening, at our feet,
With wet, entangled locks he lifeless lay.
What use to call him by his household name!
What use to press wild kisses on his brow!
An hour ago, and love or praise or blame
Had reached his ears. He does not heed them now.

He came of age! He studies God's own lore
Of heavenly wisdom and of heavenly joy;
But we—we can but feel our hearts are sore,
And that we fain once more would see our boy.
Yet know we, though our prayers could aught avail,
His was the happier fate, with so short strife
To pass beyond the impenetrable veil
Where open lies the mystery of life.—C. G. F.

